# Acute myeloid leukaemia presenting with ecthyma gangrenosum as the first manifestation: A case report

**DOI:** 10.1097/MD.0000000000025867

**Published:** 2021-05-07

**Authors:** Yoshiro Hadano, Nao Yoshida-Sakai, Yutaka Imamura, Tomohiro Inoue, Hitoshi Koga

**Affiliations:** a Department of Infectious Diseases, St. Mary's Hospital; b Biostatistics Center, Kurume University School of Medicine; c Department of Hematology; d Department of Emergency Medicine, St. Mary's Hospital, Kurume, Japan.

**Keywords:** acute myeloid leukaemia, ecthyma gangrenosum, *Pseudomonas aeruginosa*

## Abstract

**Rationale:**

Ecthyma gangrenosum (EG) is an uncommon cutaneous infection usually associated with *Pseudomonas aeruginosa* bacteremia in immunocompromised patients, particularly those with underlying malignant diseases. Despite its rarity, especially in immunocompetent or nondiagnosed immunodeficiency patients, EG can present as the first manifestation of an underlying immunosuppression.

**Patient concerns:**

A 42-year-old Japanese man was admitted to our hospital with a 3-day history of a painless red macule on his right forearm and fever.

**Diagnoses:**

Blood culture on admission revealed the presence of *Pseudomonas aeruginosa*, whereas pus culture of the skin lesion showed *Pseudomonas aeruginosa* and methicillin-susceptible *Staphylococcus aureus* positivity.

**Interventions:**

Additional bone marrow aspirate examination and immunophenotyping were performed to confirm the diagnosis of acute promyelocytic leukaemia with PML-retinoic acid alpha receptor.

**Outcomes:**

The patient was successfully treated with a 14-day course of antibiotics, and no evidence of relapse was noted. The patient achieved complete remission after treatment for acute promyelocytic leukaemia.

**Lessons:**

It should be kept in mind that EG is an important cutaneous infection that is typically associated with *P aeruginosa* bacteremia and the presence of underlying immunodeficiency, such as acute leukaemia.

## 1 Introduction

Ecthyma gangrenosum (EG) is an uncommon cutaneous infection usually associated with *Pseudomonas aeruginosa* bacteremia in immunocompromised patients, particularly those with underlying malignant diseases.^[1,2]^ Despite its rarity, especially in immunocompetent or nondiagnosed immunodeficiency patients, EG can present as the first manifestation of an underlying immunosuppression.^[3–6]^ Here, we have reported a case of acute promyelocytic leukaemia (APL) presenting with EG as the first manifestation.

## 2 Case presentation

A 42-year-old Japanese man with a history of type 2 diabetes mellitus was admitted to our hospital with a 3-day history of fever and claimed to have had insect bites for the past three weeks. Two days before admission, the patient noticed a macule with swelling on his right anterior forearm. The patient developed fever the next day, and the area of swelling on his right forearm expanded, with blisters in the center. He visited a local clinic and was prescribed cefcapene pivoxil for the diagnosis of cellulitis. However, on the day of admission, the patient developed fever, chills, and general fatigue, and the swelling on his forearm worsened. The patient returned to the local clinic and was transferred to our hospital with a diagnosis of tick bite disease.

**Figure 1:**
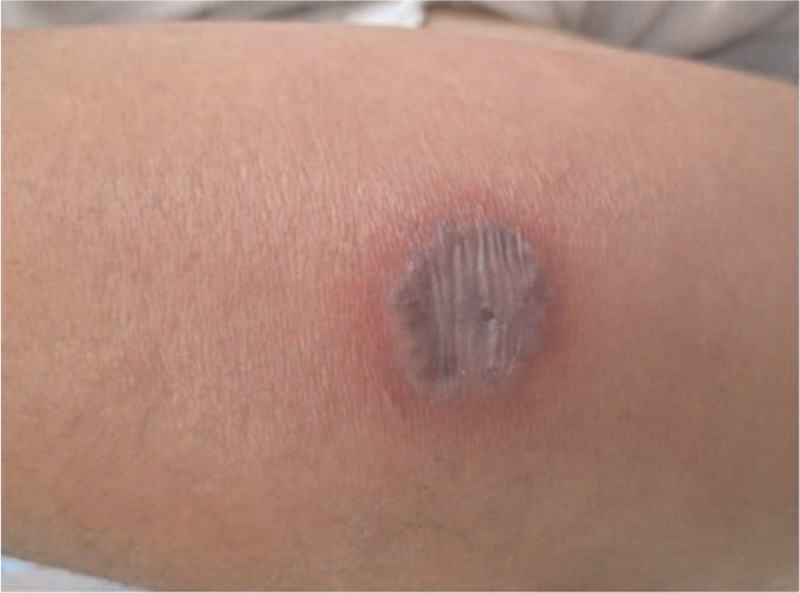
Necrotic blister measuring 20 mm in diameter on the right forearm at presentation.

He denied having sore throat, cough, dyspnea, chest pain, abdominal pain, back pain, and muscle pain. He had a history of diabetes, smoked 1 pack of cigarettes per day, and drank socially. He denied recent animal exposure, recent travel, or any history of allergies. On physical examination, his blood pressure was 149/82 mmHg, pulse rate was 101 beats per minute, body temperature was 39.4°C, respiratory rate was 22 breaths per minute, and oxygen saturation was 98% on room air. His physical examination results were unremarkable, except for tenderness and swelling on his right forearm, with a blister in the center measuring 20 mm × 20 mm (Fig. 1). Initial laboratory tests revealed a white blood cell count of 810 cells/μL with 33% neutrophils, 54% lymphocytes, 2% atypical lymphocytes, and 1% promyelocytes; hemoglobin level of 12.4 mg/dL; and platelet count of 56,000 cells/μL.

Serum chemistry evaluation revealed the following results: blood urea nitrogen 11.6 mg/dL, creatinine 0.8 mg/dL, sodium 138 mEq/L, potassium 3.7 mEq/L, chloride 92 mEq/L, albumin 4.1 g/dL, total protein 7.4 g/dL, aspartate aminotransferase 14 IU/L, alanine aminotransferase 12 IU/L, lactate dehydrogenase 189 IU/L, alkaline phosphatase 233 U/L, total bilirubin 0.5 mg/dL, glucose 208 mg/dL, C-reactive protein 23.5 mg/dL, HbA1c 6.8%, prothrombin time 16.7 seconds, activated partial thromboplastin time 36.1 seconds, fibrinogen 180 mg/dL, fibrin degradation product 416 μg/mL, and d-dimer 157 ng/mL. Urine analysis revealed normal findings, and chest radiography revealed no pneumonia.

Initial workups in the emergency room raised suspicion for a tick bite infection with cellulitis; hence, intravenous cefazolin (1 g every 8 hours) and minocycline (100 mg every 12 hours) were initiated.

On day 2, subcutaneous bleeding appeared on his left forearm, which was on the sphygmomanometer attachment site on the right upper arm. Given that his platelet count was decreased to 35,000 cells/μL, platelet transfusion (100,000 U) was performed. On day 3, his platelet count, and additional examination revealed the following results: ferritin level 2045 ng/mL (normal range, 39–340 ng/dL), and human immunodeficiency virus antibody negative. Blood culture on admission revealed *P aeruginosa*, and the lesion culture grew non-fermenters, such as gram-negative rods and *Staphylococcus*-like gram-positive cocci. Infectious diseases consultation was performed, and within 5 days the lesion developed from a macule, into a papule, and then to a hemorrhagic bulla with *P aeruginosa* bacteremia, suggestive of ecthyma gangrenosum. After repeated blood cultures, his treatment regimen was switched to cefepime (2 g every 8 hours), vancomycin (1 g every 12 hours), and minocycline (100 mg every 12 hours).

On day 4, microscopic examination of the peripheral blood smear revealed that 50% leukocytes were abnormal lymphocytes, mostly promyelocytes. Bone marrow aspirate examination and immunophenotyping confirmed the diagnosis of APL with PML-retinoic acid alpha receptor (RARA). The patient received induction chemotherapy with all-trans retinoic acid, cytarabine, and idarubicin.

On day 5, blood cultures from day 3 also revealed *P aeruginosa*, whereas pus culture grew *Pseudomonas aeruginosa* and methicillin-susceptible *Staphylococcus aureus*. The patient was continued on cefepime based on the findings of the susceptibility test, and 8 successfully treated with a 14-day course of antibiotics. IgM and IgG antibodies for *Rickettsia japonica* and severe fever with thrombocytopenia syndrome tested negative. The patient achieved complete remission after treatment for APL with PML-RARA, and no evidence of APL relapse has been noted at the 3-year follow-up. The patient is currently being monitored at our hospital.

This study was approved by the institutional review board of St. Mary's Hospital (No. 17-0203). Written consent from the patient was obtained for the purpose of publication of the case details.

## 3 Discussion

EG is a rare cutaneous infection mainly associated with *P aeruginosa*. Although EG can manifest in immunocompetent patients, the infection typically occurs in immunocompromised or critically ill patients and requires prompt diagnosis and treatment.^[1]^ Recognizing its characteristic skin appearance is the key to appropriate diagnosis and early therapy. Typically, the skin lesion initially appears as an erythematous nodule or a hemorrhagic vesicle, usually macule followed by papule, which then evolves into a necrotic ulcer with eschar.^[1]^ However, early appearance of EG is nonspecific. Common skin lesions in immunocompromised patients must be examined carefully as warning signs of impending sepsis. The buttocks or lower extremities are the common sites of the lesions, but the lesions can also present in other regions, including the face, head, and neck. In our case, the presentation of EG and bacteremia caused by *P aeruginosa* was associated with a diagnosis of APL, suggesting that EG can present as the first manifestation of an underlying immunosuppression. In addition, 4 cases of acute leukaemia presenting with EG (2 adult cases and 2 pediatric cases) have been reported in the literature.^[2–5]^ In a previous study, among patients with *P aeruginosa* EG, although most patients had underlying conditions such as leukaemia, lymphoma, other malignant diseases, or organ transplantation, approximately 40% patients were immunocompetent.^[1]^ Physicians unfamiliar with this disease can find it difficult to diagnose cases such as ours, in which the patient had not been diagnosed with any malignant diseases at the time of admission.

EG has various differential diagnoses, such as warfarin or cocaine-induced skin necrosis, calciphylaxis, septic emboli, disseminated intravascular coagulation, pyoderma gangrenosum, necrotizing fasciitis, and vasculitis.^[1]^ In our case, *P aeruginosa* and methicillin-susceptible *S aureus* were isolated from lesion culture. In a detailed analysis of 167 EG cases, 123 (73.6%) cases were associated with *P aeruginosa* infection.^[1]^ Other bacterial etiologies, including *Escherichia coli*, *S aureus*, *Aeromonas hydrophila*, and *Mucor* species, were detected in 29 (17.4%) cases.^[4–6]^ Bacteremia is relatively common in cases of *P aeruginosa* infection, but rare in cases of infection with other bacterial species.^[1]^ It was difficult to make a diagnosis in our on the first day of admission; the initial diagnosis was tick bite infection with cellulitis because there was no indication of leukemia, such as blasts on the blood smear. Given that EG can mimic tick bite infection, especially in immunocompetent or nondiagnosed immunodeficiency patients, doctors’ unfamiliarity with EG would often lead to misdiagnosis. In our case, abnormal findings of peripheral blood lymphocytes suspicious for blast cells only appeared a few days after hospitalization. Therefore, bone marrow aspiration is essential to confirm the diagnosis of leukemia. In addition, peripheral blood smears can provide a diagnostic clue, and repeat analyses are important in cases of suspected hematological disorders.

In conclusion, we have reported a case of AML presenting with EG mimicking tick bite infection. It should be kept in mind that EG is an important cutaneous infection that is typically associated with *P aeruginosa* bacteremia and the presence of underlying immunodeficiency, such as acute leukaemia, which warrants further clinical investigations.

## Acknowledgments

The authors thank the clinical staff at St. Mary's Hospital for their excellent work.

## Author contributions

**Conceptualization:** Yoshiro Hadano.

**Data curation:** Yoshiro Hadano, Nao Yoshida-Sakai

**Formal analysis:** Yoshiro Hadano

**Investigation:** Yoshiro Hadano, Nao Yoshida-Sakai, Yutaka Imamura, Tomohiro Inoue, Hitoshi Koga.

**Supervision:** Nao Yoshida-Sakai, Yutaka Imamura

**Writing – original draft:** Yoshiro Hadano

**Writing – review & editing:** Nao Yoshida-Sakai, Yutaka Imamura, Tomohiro Inoue, Hitoshi Koga
